# Spectrum of interstitial lung diseases at a tertiary center in a developing country: A study of 803 subjects

**DOI:** 10.1371/journal.pone.0191938

**Published:** 2018-02-08

**Authors:** Sahajal Dhooria, Ritesh Agarwal, Inderpaul Singh Sehgal, Kuruswamy Thurai Prasad, Mandeep Garg, Amanjit Bal, Ashutosh Nath Aggarwal, Digambar Behera

**Affiliations:** 1 Department of Pulmonary Medicine, Postgraduate Institute of Medical Education and Research, Chandigarh, India; 2 Department of Radiodiagnosis and Imaging, Postgraduate Institute of Medical Education and Research, Chandigarh, India; 3 Department of Histopathology, Postgraduate Institute of Medical Education and Research, Chandigarh, India; Vallabhbhai Patel Chest Institute, INDIA

## Abstract

**Background:**

The spectrum of interstitial lung diseases (ILDs) have mainly been reported from the developed countries; data from developing countries is sparse and conflicting. The aim of this study is to describe the distribution of various ILDs from a developing country.

**Methods:**

This is an analysis of prospectively collected clinical, radiological and histological data of consecutive subjects (age >12 years) with ILDs from a single tertiary care medical center. The diagnosis of the specific subtype of ILD was made according to standard criteria for various ILDs.

**Results:**

A total of 803 subjects (mean age, 50.6 years; 50.2% women) were enrolled between March 2015 to February 2017 of which 566 (70.5%) were diagnosed during the study period (incident cases). Sarcoidosis (42.2%), idiopathic pulmonary fibrosis (IPF, 21.2%), connective tissue disease (CTD)-related ILDs (12.7%), hypersensitivity pneumonitis (10.7%), and non-IPF idiopathic interstitial pneumonias (9.2%) were the most common ILDs. The spectrum of ILDs was not significantly different (p = 0.87) between incident and prevalent cases. A histopathological specimen was obtained in 49.9% of the subjects yielding a histologically confirmed diagnosis in 40.6%. A diagnostic procedure was not performed in 402 subjects; the most common reasons were presence of definite usual interstitial pneumonia pattern on high resolution computed tomography and patients’ unwillingness to undergo the procedure.

**Conclusion:**

Sarcoidosis, IPF and CTD-ILDs were the most common ILDs seen at a tertiary center in northern India similar to the spectrum reported from developed countries. More studies are required from developing countries to ascertain the spectrum of ILDs in different geographic locales.

## Introduction

Interstitial lung diseases (ILDs) or diffuse parenchymal lung diseases are a heterogeneous group of disorders characterized by varying degrees of inflammation and fibrosis in the lung parenchyma.[1] Lately, there has been an exponential increase in the understanding of various ILDs. It is essential to differentiate between these various disease entities, as there are significant differences amongst them in the risk factors, pathogenesis, treatment and outcomes.[2]

Several studies from across the globe have reported on the incidence, prevalence and the relative frequency of ILDs.[3–7] The annual incidence of ILDs has variably been reported between 1 and 31.5 per 100,000.[3, 4, 6–11] Unfortunately, a large number of these studies have not used the classification proposed by the 2002 American Thoracic Society (ATS)/European Respiratory Society (ERS) consensus statement on idiopathic interstitial pneumonias (IIPs), which is now considered a benchmark.[3, 4, 6–10, 12–15] Also, a majority of the studies have been performed in the developed countries (Europe and North America). With differences in the genetic profile, environmental factors, occupational exposures, smoking habits, socio-cultural and farming practices in developing countries, the spectrum of ILDs may be different from other regions of the world.[16, 17] There is an unmet need for studies on the epidemiology of ILDs from the developing world. Although, there are a few studies from developing countries that have reported the case-mix of ILDs from tertiary centers, majority of these studies were small and have not used the standard criteria for the diagnosis of various ILDs. [15, 18–22]

Recently, a large multicenter registry from India has reported hypersensitivity pneumonitis (HP) as the most prevalent ILD, contrary to other data from the same country and other geographic regions.[15] Herein, we report the spectrum of ILDs from a tertiary care center in northern India. We also analyze the similarities and differences of the profile of ILDs from other such studies reported from India and worldwide.

## Materials and methods

This was an analysis of data prospectively collected over two years (between March 2015 and February 2017) in the Chest Clinic of this Institute. The Institute Ethics Committee approved the study protocol, and a written informed consent was obtained from all subjects.

### Subjects and study procedures

All patients referred to the Chest Clinic with a diagnosis of ILD were included in the study. A detailed history was obtained with regards to the risk factors for various ILDs including presence of a connective tissue disease (CTD), drug and environmental exposures. The following set of investigations was obtained on the basis of the suspected diagnosis: chest radiograph, high resolution computed tomography (HRCT) of the thorax, spirometry, serology for autoimmune diseases, and serum angiotensin convertase enzyme (ACE) levels. Subjects also underwent one or more of the following investigations to obtain a pathological diagnosis: bronchoalveolar lavage, transbronchial needle aspiration (conventional or endobronchial ultrasound-guided), endobronchial biopsy, transbronchial lung biopsy (TBLB) (performed using conventional forceps or with a cryoprobe [cryo-TBLB]), surgical lung biopsy, fine needle aspiration, biopsy of any other involved site such as skin, as required. For subjects who had undergone diagnostic evaluation before the start of the study period, all available data were recorded.

### Diagnosis of ILDs

For the diagnosis of IPF, the ATS/ERS/Japanese Respiratory Society/Latin American Thoracic Association guidelines were followed.[23] For the diagnosis of other IIPs, the ATS/ERS Multidisciplinary Consensus Classification of the IIPs was followed.[1, 2] A diagnosis of sarcoidosis was made on the basis of consistent clinical and radiological findings, and the presence of granulomatous inflammation in tissue specimens, in the absence of other known causes such as tuberculosis.[24–26] If granulomatous inflammation could not be demonstrated, the diagnosis of sarcoidosis was made after a follow up of six months. A diagnosis of HP was made based on a history of exposure to organic dusts, typical HRCT appearance (any combination of ground glass opacities, ground glass centrilobular nodules, septal thickening, mosaic attenuation and honeycombing), along with histological findings of HP on lung biopsy. A diagnosis of a CTD related ILD was made in the presence of a CTD (rheumatoid arthritis, systemic sclerosis, and others) and the presence of ILD on HRCT of the chest. The subjects were evaluated by a rheumatologist and a diagnosis of CTD was made based on standard criteria. A diagnosis of interstitial pneumonia with autoimmune features (IPAF) was made using the ATS/ERS research statement.[27]

All clinical, radiologic, and histopathologic data were reviewed by a multidisciplinary team comprising of pulmonologists (with expertise in ILDs) along with a dedicated pulmonary radiologist and a pulmonary pathologist. In case, a biopsy was not performed, a best fit diagnosis was made on the basis of clinical details, HRCT findings and findings on ancillary investigations (such as ACE levels and autoantibodies). In case, all the available information did not suggest a particular type of ILD, a diagnosis of unclassifiable ILD was made. Subjects who were diagnosed during the study period were termed as incident cases. Subjects who were diagnosed before or during the study period were termed as prevalent cases.

### Statistical analysis

Data were analyzed using the statistical package SPSS (SPSS version 22, for Windows; IBM SPSS Inc., Chicago, IL). Data are expressed as mean ± standard deviation (SD), median (interquartile range), or as number (percentage). Categorical data were compared using the chi-square test. A p value of <0.05 was considered as statistically significant.

## Results

A total of 803 subjects (mean [SD] age, 50.6 [13.8] years, 403 [50.2%] women) were enrolled during the study period. The baseline characteristics of the study subjects are shown in Table 1. Cough was the most common symptom (86.1%) followed by breathlessness (76.1%), weight loss (30.9%), anorexia (24.2%), joint pains (23.9%), and fatigue (17.9%). Most (58.6%) subjects had a restrictive defect on spirometry. The most common abnormalities on HRCT chest were interlobular septal thickening (43.7%), intralobular septal thickening (39.2%), ground glass opacities (31.6%), and honeycombing (30.3%). Mediastinal lymphadenopathy was present in 336 (47.4%) subjects.

**Table 1:** Baseline characteristics of the study subjects (n = 803).

Characteristic	Value
Age, years	50.6 ± 13.8
Women	403 (50.2)
Body mass index (kg/m^2^)	25.5 ± 4.6
Smokers	110 (13.7)
History of tuberculosis	148 (18.4)
Duration of symptoms at diagnosis	6 (3–10)
Duration of follow up of prevalent cases in months, median (IQR)	22 (10.0–48.0)
Area of residence	
Rural	221 (27.5)
Urban	582 (72.5)
Occupation	
Office-based	125 (17.4)
Private enterprise	54 (7.5)
Farmer	94 (13.1)
Medical-Paramedical	22 (3.1)
Homemaker	290 (40.3)
Teacher	36 (5.0)
Others	98 (13.6)
Spirometric abnormality	
Normal	215 (32.4)
Obstruction	60 (9.0)
Restriction	389 (58.6)
Spirometric measurements	
FVC	2.30 ± 0.93
FVC %predicted	72.5 ± 20.7
FEV1	1.85 ± 0.75
FEV1%predicted	74.7 ± 20.9
FEV1/FVC ratio	0.81 ± 0.09
Oxygen saturation at rest, median (IQR)	98 (95–98)
Abnormalities on HRCT of the chest	
Interlobular septal thickening	351 (43.7)
Intralobular septal thickening	315 (39.2)
Peribronchovascular septal thickening	198 (24.7)
Random nodules	42 (5.2)
Centrilobular nodules	19 (2.4)
Mosaic attenuation	52 (6.5)
Ground glass opacities	254 (31.6)
Honeycombing	243 (30.3)
Mediastinal lymph nodes	336 (47.4)
Cysts	21 (2.6)
Consolidation	46 (5.7)
Distribution of abnormalities on HRCT of the chest	
Upper/middle lobe predominant	174 (21.7)
Lower lobe predominant	277 (34.5)
Diffuse	249 (31.0)

FEV1-forced expiratory volume in one second, FVC-forced vital capacity, HRCT-high resolution computed tomography; IQR- interquartile range. All values are mean ± standard deviation or number percentage, unless otherwise specified.

Sarcoidosis was the most common (42.2%) ILD (Table 2), followed by IPF (21.2%). CTD-ILDs, HP, and non-IPF IIPs were diagnosed in 12.7%, 10.7%, and 9.2% of the subjects, respectively. Most (63.4%) subjects with sarcoidosis had stage II or III disease. The mean forced vital capacity of subjects with sarcoidosis was higher than those with other diseases. Subjects with IPF were predominantly males (71%) and older in age than those with other diagnoses (Table 3). Rheumatoid arthritis and systemic sclerosis associated ILDs were the most common CTD-ILDs identified. A total of 86 patients were diagnosed to have HP. In most patients (59.3%), the disease was attributable to farm dust exposure. Exposure to bird feathers and excreta was present in 15.1% of the subjects with HP. In 19.8% of the subjects, the environmental exposure causing the disease remained unknown. Of the 86 subjects with HP, 30 had undergone a lung biopsy, of whom 21 had a histological findings diagnostic or suggestive of HP. Idiopathic nonspecific interstitial pneumonia was the most common non-IPF IIP encountered; other IIPs were rarely encountered.

**Table 2:** Final diagnoses of study subjects.

Diagnosis	Number (percentage)
Sarcoidosis	339 (42.2)
Stage 0	17 (5.0)
Stage I	96 (28.3)
Stage II	138 (40.7)
Stage III	77 (22.7)
Stage IV	11 (3.2)
Idiopathic pulmonary fibrosis (IPF)	170 (21.2)
Non-IPF idiopathic interstitial pneumonia	74 (9.2)
Nonspecific interstitial pneumonia	63 (7.8)
Acute Interstitial Pneumonia	2 (0.2)
Cryptogenic Organizing Pneumonia	4 (0.5)
Respiratory Bronchiolitis-ILD/Desquamative Interstitial Pneumonia	5 (0.6)
Connective tissue disease associated ILD	102 (12.7)
Rheumatoid arthritis	22 (2.7)
Systemic sclerosis	19 (2.3)
Mixed connective tissue disease	4 (0.5)
Sjogren’s syndrome	3 (0.4)
Systemic lupus erythematosus	3 (0.4)
Dermatomyositis/Anti-synthetase syndrome	6 (0.7)
Interstitial pneumonia with autoimmune features	45 (5.6)
Hypersensitivity pneumonitis	86 (10.7)
Farmer’s lung	51 (6.4)
Bird fancier’s lung	13 (1.6)
Miller’s lung	3 (0.4)
Other exposures	2 (0.2)
Unknown exposure	17 (2.1)
Drug-induced ILD	6 (0.7)
Bleomycin	4 (0.5)
Methotrexate	2 (0.3)
Occupational lung disease	7 (0.9)
Arc welder’s lung	1 (0.1)
Metal worker’s lung	1 (0.1)
Silicosis	4 (0.5)
Pneumoconiosis, NOS	1 (0.1)
Unclassifiable	7 (0.9)
Others	12 (1.5)
Chronic eosinophilic pneumonia	2 (0.2)
Cystic lung disease, NOS	1 (0.1)
CVID associated LIP	1 (0.1)
IgG4 associated fibrosis	2 (0.2)
Pulmonary alveolar microlithiasis	1 (0.1)
Pulmonary alveolar proteinosis	2 (0.2)
Lymphangiolieomyomatosis	1 (0.1)
Idiopathic pulmonary hemosiderosis	1 (0.1)
Pulmonary Langerhan’s cell histiocytosis	1 (0.1)

CVID-common variable immunodeficiency, ILD-interstitial lung disease, LIP-lymphocytic interstitial pneumonia, NOS-not otherwise specified.

**Table 3:** Baseline characteristics of subjects with different diagnoses.

	All (n = 803)	Sarcoidosis (n = 339)	IPF (n = 170)	Non-IPF IIP (n = 74)	CTD-ILD (n = 102)	HP (n = 86)	Others (n = 32)
Age	50.6±13.8	44.8±11.8	64.4±9.4	52.8±10.4	49.0±11.9	47.6±13.9	46.5±13.5
Gender, men	400 (49.8)	166 (49.0)	120 (70.6)	24 (32.4)	26 (25.5)	44 (51.2)	20 (62.5)
Smokers	110 (13.7)	17 (5.0)	74 (43.5)	7 (9.5)	4 (3.9)	6 (7.0)	2 (6.3)
History of tuberculosis	148 (18.4)	68 (20.1)	29 (17.1)	8 (10.8)	15 (14.7)	22 (25.6)	6 (18.8)
Symptom duration at presentation	6 (3–10)	5 (3–8)	6 (4–12)	6 (4–8)	6 (4–10)	8 (5–24)	6 (2–12)
Duration of follow up among prevalent cases	22 (10–48)	23 (12–49)	11 (5–27)	49 (15–85)	25 (9–45)	21 (11–46)	29 (8–35)
FVC	2.3±0.9	2.8±0.9	2.1±0.7	1.8±0.6	1.8±0.6	1.9±0.9	2.3±1.0
FVC, % predicted	72±21	83±19	67±18	62±17	63±18	60±19	66±22
Oxygen saturation at rest	96±4	97±2	94±5	95±6	96±3	95±4	94±8
Distribution of abnormalities on HRCT							
Upper lobe predominant	174 (21.7)	121 (35.7)	2 (1.2)	0	4 (3.9)	42 (48.8)	5 (15.6)
Lower lobe predominant	277 (34.5)	22 (6.5)	138 (81.2)	41 (55.4)	70 (68.6)	2 (2.3)	4 (12.5)
Diffuse	249 (31.0)	94 (27.7)	30 (17.6)	33 (44.6)	28 (27.5)	41 (47.7)	23 (71.9)
Subjects who underwent biopsy	401 (49.9)	301 (88.8)	10 (5.9)	27 (36.5)	14 (13.7)	30 (34.9)	19 (59.4)
Subjects with a diagnostic/contributory biopsy	359 (44.7)	288 (84.9)	4 (2.4)	22 (29.7)	10 (9.8)	21 (24.4)	14 (43.8)

CTD-connective tissue disease, FVC-forced vital capacity, HP-hypersensitivity pneumonitis, HRCT-high resolution computed tomography, IIP-idiopathic interstitial pneumonia, ILD-interstitial lung disease, IPF-idiopathic pulmonary fibrosis. All values are mean±standard deviation, median (interquartile range) or number (percentage).

Four hundred and one (49.9%) subjects underwent a procedure for obtaining a cytological/histological diagnosis. Of these, 326 (81.3%) specimens were diagnostic, thus yielding a histologically confirmed disease in 40.6% of the total subjects (Table 4). In 33 (8.2%) subjects with non-diagnostic biopsy, the histopathology contributed significantly to the final multidisciplinary diagnosis. A biopsy was not performed in 402 subjects (50.1%), the most common reason (35.3%) for not performing a lung biopsy was a definite pattern of usual interstitial pneumonia on HRCT chest (Table 5). A significant proportion of these subjects (89/402, 22.1%) were not willing to undergo a biopsy procedure.

**Table 4:** Details of procedures for cytological/histological diagnoses of ILDs in study subjects (n = 401).

	Diagnostic	Non-diagnostic but contributing important information to MDD	Non-diagnostic	Total number
Transbronchial lung biopsy	42 (48.8)	27 (31.4)	17 (19.8)	86
Any combination of transbronchial lung biopsy, endobronchial biopsy and transbronchial needle aspiration	255 (92.4)	2 (0.7)	19 (6.9)	276
Transbronchial lung cryobiopsy	14 (58.3)	4 (16.7)	6 (25)	24
Surgical lung biopsy	5 (100)	0	0	5
Other diagnostic procedures	10 (100)	0	0	10
Total	326 (81.3)	33 (8.2)	42 (10.5)	401

MDD- multidisciplinary discussion. Other diagnostic procedures included skin biopsy, liver biopsy, fine needle aspiration from lymph nodes and spleen, bronchoalveolar lavage and computed tomography guided lung biopsy.

**Table 5:** Reasons for not obtaining a histological diagnosis (n = 402).

Reason	Number (percentage)
Definite UIP on CT	142 (35.3)
Definite CT appearance of other conditions	21 (5.2)
CTD-ILD	59 (14.7)
IPAF	23 (5.7)
Patient unfit to undergo procedure	39 (9.7)
Patient unwilling for procedure	89 (22.1)
Histopathological details not available	29 (7.2)

CT-computed tomography, CTD-connective tissue disease, ILD-interstitial lung disease, IPAF-interstitial pneumonia with autoimmune features, UIP-usual interstitial pneumonia

Of the total 803 subjects, 566 subjects were diagnosed within the study period and were termed as incident cases, while the entire cohort was labelled as prevalent cases. The spectrum of ILDs was not significantly different (p = 0.87) between incident and prevalent cases (Table 6).

**Table 6:** Comparison of diagnosis of incident and prevalent cases.

Diagnosis	Incident (n = 566)	Prevalent (n = 803)
Sarcoidosis	217 (38.3)	339 (42.2)
Idiopathic pulmonary fibrosis	130 (23.0)	170 (21.2)
Non-IPF IIP	47 (8.3)	74 (9.2)
Connective tissue disease related ILD	77 (13.6)	102 (12.7)
Hypersensitivity pneumonitis	69 (12.2)	86 (10.7)
Drug induced lung disease	5 (0.9)	6 (0.7)
Occupational lung disease	6 (1.1)	7 (0.9)
Others	15 (2.7)	19 (2.4)

IIP-idiopathic interstitial pneumonia, ILD-interstitial lung disease, IPF-idiopathic pulmonary fibrosis.

## Discussion

The results of this study suggest that sarcoidosis (42%) and IPF (21%) are the most common ILDs in patients presenting to a tertiary care center in northern India. To our knowledge, this is the largest single center experience of spectrum of ILDs reported till date.

The overall spectrum of ILDs reported in this study is similar to that reported from several multi-center registries from Europe (Table 7). In most of these large studies, sarcoidosis and IPF have been identified as the most prevalent forms of ILDs.[4, 6, 7, 9, 12] However, there are subtle differences. Our prevalence of sarcoidosis (42%) is similar to that reported from Germany (45%), Turkey (37%) and a previous study from India (37%).[5, 11, 18] However, it is substantially higher than that reported from the United States, Belgium, Spain, Italy, and Greece (12–34%).[3, 4, 6–9, 13, 15] The frequency of IPF (21%) and non-IPF IIPs (9%) is similar to that reported from Greece (20% and 10%, respectively), and Saudi Arabia (23% and 9%) but lower than the combined frequencies (of IPF and non-IPF IIPs) reported in earlier studies (35–43%), prior to 2002.[5, 9, 12, 13] A significant proportion of the ILDs in our study (13%) are related to CTDs, identical to the findings from India and other countries (12–14%).[3, 9, 10] The similarity of the spectrum of the ILDs to that reported from developed countries may be due to reasons such as the predominantly urban population in the current study, relatively little mining and other major industrial activity in our region, and possibly, due to the referral pattern of local physicians. The differences in the profile of ILDs as compared to other studies from India may be attributable to differences in the genetic, ecological and exposure profiles in different regions of the country (Table 7).[15, 18, 19, 21]

**Table 7:** Spectrum of diffuse parenchymal lung diseases in previous studies.

	Country	Number	Incident/prevalent	Sarcoidosis, %	IPF, %	Non IPF IIPs, %	CTD-ILD, %	HP, %	Drug/radiation induced ILD, %	Occupational lung disease, %	Unclassifiable, %	Others, %
Coultas et al. (1994)[3]	US	258	Prevalent	11.6	22.5		12.8	0	2.3	13.9	30.9	6.0
		202	Incident	7.8	31.7		9.0	1.5	5.0	10.4	29.6	5.5
Schweisfurth et al. (1996)[12]	Germany	234	Prevalent	35.4	39.2		2.1	13.2	2.5	2.6	5.1	-
Agostini et al. (2001)[13]	Italy	1382	Prevalent	29.2	43.4		-	3.7	1.7	-	-	8.5
Thomeer et al. (2001)[14]	Belgium	362	Prevalent	31	20		7	13	3	6	9	11
		264	Incident	26	22		7	12	5	7	10	11
Schweisfurth et al. (2003)[5]	Germany	1142	Prevalent	44.7	35.1		1.8	12.7	0.4	3.2		2.0
Lopez-Campos et al. (2004)[8]	Spain	744	Incident	11.7	38.6		6.0	5.1	2.4	7.4	9.3	18.9
Xaubet et al. (2004)[6]*	Spain	511	Incident	14.9	38.6	13.8	10.0	6.6	4.1	-	5.1	6.8
Tinelli et al. (2005)[7]*	Italy	3152	Prevalent	33.7	27.4	11.6	-	2.9	1.2	-	-	5.3
Karakatsani et al. (2009)[9]*	Greece	967	Prevalent	34.1	19.5	10.0	12.4	2.6	1.8	2.0	8.5	9.1
Sen et al. (2010)[19]	India	274	Prevalent	22	46.7		18	6	1.1	0.7	-	4.7
Alhamad et al. (2013)[20]*	Saudi Arabia	330	Incident	20	23.3	9.0	34.8	6.4	1.2	-	1.8	2.7
Hyldgaard et al. (2014)[10]*	Denmark	431	Incident	NI	28	16	13	7	5	-	25	4
Musellim et al. (2014)[11]*	Turkey	2245	Incident	37.6	19.9	6.1	9.8	4.0	3.5	11.8	-	7.2
Rajkumar et al. (2014)[18]*	India	289	Prevalent	37.4	27.7	27.3	4.5	2.4	-	-	-	0.7
Singh et al. (2016)[15]*	India	1084	Incident	7.8	13.7	12.4	13.9	47.3	0.3	3.0	0.2	1.5
Present study*	India	803	Prevalent	42.2	21.2	9.2	12.7	10.7	0.7	0.9	0.9	1.5

*Studies that followed the 2002 American Thoracic Society/European Respiratory Society consensus criteria for classification. CTD-connective tissue disease, HP-hypersensitivity pneumonitis, IIP-idiopathic interstitial pneumonia, ILD-interstitial lung disease, IPF-idiopathic pulmonary fibrosis, NI-not included.

**Fig 1:**
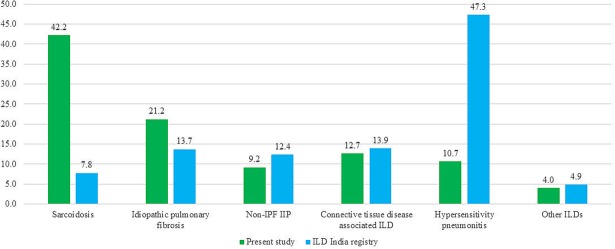
Comparison of spectrum of interstitial lung diseases in this study and a recently reported multicenter study from India. [15] The numbers represent percentage of subjects diagnosed with the condition.

Our results are in striking contrast to those of a recently published multicenter registry of ILDs from India involving 1,084 patients (Fig 1).[15] In this study, HP accounted for 47.3% of all ILDs, IPF was diagnosed in 13.7% while sarcoidosis was encountered in a only 7.8% of the subjects. This registry data from India suffers from several limitations.[28–30] Subjects were enrolled non-consecutively from small hospitals and individual clinics in scattered locations leading to an important selection bias. A lung biopsy was performed in merely 7.5% of the subjects. Most importantly, a diagnosis of aircooler-induced HP was made untenably, in an astonishingly high proportion of patients (48.1% of all patients with HP), without establishing a cause-and-effect relationship. In contrast to this study by Singh et al., HP was found in 10.7% of the patients in the present study. This proportion is similar to that reported from centers in Germany (13.2%), Belgium (13%), and an earlier study from India (11%).[21] while it is higher than that reported from the United States, Italy, Greece, Denmark and other studies from India (1.5–7%).[3, 4, 7, 9, 10, 12, 18, 19] This variation in the frequency of HP across different geographic locations can be attributed to differences in farming practices and hobbies such as bird keeping across various regions in the world and in India. Further, the diagnostic criteria followed for the diagnosis of various ILDs across different studies were not uniform.

About 50% of our patients underwent some form of cytological/ histopathological diagnostic procedure. A histological confirmation was achieved in about 41% of the subjects, comparable to previous studies.[3, 4, 6, 8] In the current study, a biopsy was not required in most of the IPF patients because of a typical UIP pattern on HRCT in 142/170 (84%) of the patients (Table 5). We did not perform lung biopsy in most patients with a definite CTD (n = 59) or those with IPAF (n = 23), as we felt that the ultimate diagnosis on biopsy would not change the management of these patients significantly, as observed previously.[4, 31] We attempted to obtain a lung biopsy in most of our patients suspected to have a non-IPF IIP, HP, or other ILDs. However, a significant proportion of our patients (128/803 [15.9%]) did not undergo the procedure as they were either unwilling or unfit for the procedure. Due to the unavailability of video-assisted thoracic surgery (VATS) at our center, we were able to offer only open lung biopsy. Recently, we have also started performing transbronchial lung cryobiopsy (TBLC) for the diagnosis of ILD, with a subsequent improvement in our biopsy rates.[32, 33] In a multicenter study that included our center, the diagnostic yield of TBLC in diffuse lung disease was 78.1%.[34] In a recent systematic review, it was found that the procedure offers a fair yield of about 86% with acceptable complication rates.[35] However, the overall proportion of histologically confirmed disease in subjects with a diagnosis other than sarcoidosis was low in the present study and is certainly an important limitation.

What are the implications of this study? The present time is a very important juncture in the field of ILDs. There is a call for establishing multinational registries for various ILDs including IPF.[36] Several clinical trials on a disease such as IPF, which was considered too rare a disease for performing meaningful intervention studies, have been conducted by involving multiple centers across the world.[37, 38] Thus, it is very important to know the epidemiology of ILDs from large referral centers from across the world, including developing countries so that the burden of the various disease entities can be calculated and collaborative studies on the management of various ILDs can be planned. The knowledge of physicians regarding IPF and other ILDs has been found lacking in our region similar to other regions of the world.[39] Epidemiological information especially regarding disease burden and spectrum help to sensitize the primary care physicians to these diseases, and helps in influencing policymaking and advocacy activities.

The present study has some other limitations. This is a single center study, and thus gives an estimate of the epidemiology of ILDs from only a single region of the country. Single center studies are fraught with demographic, economic and social biases. The only advantage that a single referral center study may offer over studies from multiple primary care centers, especially for ILDs is that the diagnostic procedures and algorithms are more uniform and protocolized. However, the study population may not be representative of patients with ILD in other regions of India or other developing countries. This study predominantly included subjects residing in urban areas. Therefore, generalization of the results from this study to other regions in this country or elsewhere should be made cautiously. The biopsy rate was low (about 50%), although we attempted to obtain a lung biopsy, wherever feasible. Serologic testing for HP was not available. Nevertheless, the study offers the largest single center experience on the spectrum of ILDs encountered in clinical practice.

## Conclusions

In conclusion, the spectrum of ILDs at a tertiary center in northern India was found to be comparable to the previously reported experience from the developed countries of Europe and Northern America. Further studies are required from different regions of the world, so that the global burden of ILDs can be defined accurately. Future studies should follow stringent criteria for diagnosis of ILDs.

A histological confirmation of the disease should be attempted wherever possible and should always be combined with a multidisciplinary discussion.

## References

[1] American Thoracic Society/European Respiratory Society International Multidisciplinary Consensus Classification of the Idiopathic Interstitial Pneumonias. This joint statement of the American Thoracic Society (ATS), and the European Respiratory Society (ERS) was adopted by the ATS board of directors, June 2001 and by the ERS Executive Committee, June 2001. Am J Respir Crit Care Med. 2002;165(2):277–304. Epub 2002/01/16. doi: 10.1164/ajrccm.165.2.ats01 . 11790668 10.1164/ajrccm.165.2.ats01

[2] Travis WD, Costabel U, Hansell DM, King TE Jr., Lynch DA, Nicholson AG, et al An official American Thoracic Society/European Respiratory Society statement: Update of the international multidisciplinary classification of the idiopathic interstitial pneumonias. Am J Respir Crit Care Med. 2013;188(6):733–48. Epub 2013/09/17. doi: 10.1164/rccm.201308-1483ST . 24032382 10.1164/rccm.201308-1483ST PMC5803655

[3] Coultas DB, Zumwalt RE, Black WC, Sobonya RE. The epidemiology of interstitial lung diseases. Am J Respir Crit Care Med. 1994;150(4):967–72. Epub 1994/10/01. doi: 10.1164/ajrccm.150.4.7921471 . 7921471 10.1164/ajrccm.150.4.7921471

[4] Thomeer M, Demedts M, Vandeurzen K. Registration of interstitial lung diseases by 20 centres of respiratory medicine in Flanders. Acta Clin Belg. 2001;56(3):163–72. Epub 2001/08/04. doi: 10.1179/acb.2001.026 . 11484513 10.1179/acb.2001.026

[5] Schweisfurth H, Kieslich C, Satake N, Loddenkemper R, Schonfeld N, Mader I, et al [How are interstitial lung diseases diagnosed in Germany? Results of the scientific registry for the exploration of interstitial lung diseases ("Fibrosis registry") of the WATL]. Pneumologie. 2003;57(7):373–82. Epub 2003/07/16. doi: 10.1055/s-2003-40557 . 12861493 10.1055/s-2003-40557

[6] Xaubet A, Ancochea J, Morell F, Rodriguez-Arias JM, Villena V, Blanquer R, et al Report on the incidence of interstitial lung diseases in Spain. Sarcoidosis Vasc Diffuse Lung Dis. 2004;21(1):64–70. Epub 2004/05/07. . 15127977

[7] Tinelli C, De Silvestri A, Richeldi L, Oggionni T. The Italian register for diffuse infiltrative lung disorders (RIPID): a four-year report. Sarcoidosis Vasc Diffuse Lung Dis. 2005;22 Suppl 1:S4–8. Epub 2006/02/07. . 16457011

[8] Lopez-Campos JL, Rodriguez-Becerra E. Incidence of interstitial lung diseases in the south of Spain 1998–2000: the RENIA study. Eur J Epidemiol. 2004;19(2):155–61. Epub 2004/04/13. . 15074571 10.1023/b:ejep.0000017660.18541.83

[9] Karakatsani A, Papakosta D, Rapti A, Antoniou KM, Dimadi M, Markopoulou A, et al Epidemiology of interstitial lung diseases in Greece. Respir Med. 2009;103(8):1122–9. Epub 2009/04/07. doi: 10.1016/j.rmed.2009.03.001 . 19345567 10.1016/j.rmed.2009.03.001

[10] Hyldgaard C, Hilberg O, Muller A, Bendstrup E. A cohort study of interstitial lung diseases in central Denmark. Respir Med. 2014;108(5):793–9. Epub 2014/03/19. doi: 10.1016/j.rmed.2013.09.002 . 24636811 10.1016/j.rmed.2013.09.002

[11] Musellim B, Okumus G, Uzaslan E, Akgun M, Cetinkaya E, Turan O, et al Epidemiology and distribution of interstitial lung diseases in Turkey. Clin Respir J. 2014;8(1):55–62. Epub 2013/05/29. doi: 10.1111/crj.12035 . 23711298 10.1111/crj.12035

[12] Schweisfurth H. [Report by the Scientific Working Group for Therapy of Lung Diseases: German Fibrosis Register with initial results]. Pneumologie. 1996;50(12):899–901. Epub 1996/12/01. . 9091884

[13] Agostini C, Albera C, Bariffi F, De Palma M, Harari S, Lusuardi M, et al First report of the Italian register for diffuse infiltrative lung disorders (RIPID). Monaldi Arch Chest Dis. 2001;56(4):364–8. Epub 2002/01/05. . 11770220

[14] Thomeer MJ, Costabe U, Rizzato G, Poletti V, Demedts M. Comparison of registries of interstitial lung diseases in three European countries. Eur Respir J Suppl. 2001;32:114s–8s. Epub 2002/01/31. . 11816817

[15] Singh S, Collins BF, Sharma BB, Joshi JM, Talwar D, Katiyar S, et al Interstitial Lung Disease in India. Results of a Prospective Registry. Am J Respir Crit Care Med. 2017;195(6):801–13. Epub 2016/09/30. doi: 10.1164/rccm.201607-1484OC ; PubMed Central PMCID: PMC27684041. 27684041 10.1164/rccm.201607-1484OC

[16] Jindal SK, Aggarwal AN, Gupta D. Dust-induced interstitial lung disease in the tropics. Curr Opin Pulm Med. 2001;7(5):272–7. Epub 2001/10/05. . 11584175 10.1097/00063198-200109000-00004

[17] Jindal SK, Gupta D, Aggarwal AN. Treatment issues in interstitial lung disease in tropical countries. Curr Opin Pulm Med. 1999;5(5):287–92. Epub 1999/08/26. . 10461532 10.1097/00063198-199909000-00004

[18] Kumar R, Gupta N, Goel N. Spectrum of interstitial lung disease at a tertiary care centre in India. Pneumonol Alergol Pol. 2014;82(3):218–26. Epub 2014/05/06. doi: 10.5603/PiAP.2014.0029 . 24793149 10.5603/PiAP.2014.0029

[19] Sen T, Udwadia ZF. Retrospective study of interstitial lung disease in a tertiary care centre in India. Indian J Chest Dis Allied Sci. 2010;52(4):207–11. Epub 2011/02/10. . 21302597

[20] Alhamad EH. Interstitial lung diseases in Saudi Arabia: A single-center study. Ann Thorac Med. 2013;8(1):33–7. Epub 2013/02/27. doi: 10.4103/1817-1737.105717 ; PubMed Central PMCID: PMCPMC3573556. 23440334 10.4103/1817-1737.105717 PMC3573556

[21] Kundu S, Mitra S, Ganguly J, Mukherjee S, Ray S, Mitra R. Spectrum of diffuse parenchymal lung diseases with special reference to idiopathic pulmonary fibrosis and connective tissue disease: An eastern India experience. Lung India. 2014;31(4):354–60. Epub 2014/11/08. doi: 10.4103/0970-2113.142115 ; PubMed Central PMCID: PMCPMC4220317. 25378843 10.4103/0970-2113.142115 PMC4220317

[22] Maheshwari U, Gupta D, Aggarwal AN, Jindal SK. Spectrum and diagnosis of idiopathic pulmonary fibrosis. Indian J Chest Dis Allied Sci. 2004;46(1):23–6. Epub 2004/02/12. . 14870865

[23] Raghu G, Collard HR, Egan JJ, Martinez FJ, Behr J, Brown KK, et al An official ATS/ERS/JRS/ALAT statement: idiopathic pulmonary fibrosis: evidence-based guidelines for diagnosis and management. Am J Respir Crit Care Med. 2011;183(6):788–824. Epub 2011/04/08. doi: 10.1164/rccm.2009-040GL . 21471066 10.1164/rccm.2009-040GL PMC5450933

[24] Statement on sarcoidosis. Joint Statement of the American Thoracic Society (ATS), the European Respiratory Society (ERS) and the World Association of Sarcoidosis and Other Granulomatous Disorders (WASOG) adopted by the ATS Board of Directors and by the ERS Executive Committee, February 1999. Am J Respir Crit Care Med. 1999;160(2):736–55. Epub 1999/08/03. doi: 10.1164/ajrccm.160.2.ats4-99 . 10430755 10.1164/ajrccm.160.2.ats4-99

[25] Dhooria S, Agarwal R, Aggarwal AN, Bal A, Gupta N, Gupta D. Differentiating tuberculosis from sarcoidosis by sonographic characteristics of lymph nodes on endobronchial ultrasonography: A study of 165 patients. J Thorac Cardiovasc Surg. 2014;148(2):662–7. Epub 2014/02/19. doi: 10.1016/j.jtcvs.2014.01.028 . 24534680 10.1016/j.jtcvs.2014.01.028

[26] Dhooria S, Gupta N, Bal A, Sehgal IS, Aggarwal AN, Sethi S, et al Role of Xpert MTB/RIF in differentiating tuberculosis from sarcoidosis in patients with mediastinal lymphadenopathy undergoing EBUS-TBNA: a study of 147 patients. Sarcoidosis Vasc Diffuse Lung Dis. 2016;33(3):258–66. Epub 2016/10/21. . 27758992

[27] Fischer A, Antoniou KM, Brown KK, Cadranel J, Corte TJ, du Bois RM, et al An official European Respiratory Society/American Thoracic Society research statement: interstitial pneumonia with autoimmune features. Eur Respir J. 2015;46(4):976–87. Epub 2015/07/15. doi: 10.1183/13993003.00150-2015 . 26160873 10.1183/13993003.00150-2015

[28] Dhooria S, Agarwal R, Sehgal IS, Aggarwal AN, Behera D. The ILD-India Registry: Ignoratio Elenchi. Am J Respir Crit Care Med. 2017;195(6):835–6. Epub 2017/03/16. doi: 10.1164/rccm.201610-2023LE . 28294650 10.1164/rccm.201610-2023LE

[29] Madan K, Hadda V, Mohan A, Guleria R. The ILD-India Registry: Look Before You Leap. Am J Respir Crit Care Med. 2017;195(6):836–7. Epub 2017/03/16. doi: 10.1164/rccm.201610-2099LE . 28294659 10.1164/rccm.201610-2099LE

[30] Udwadia ZF, Richeldi L. Interstitial Lung Disease in India. Keep Searching and You'll Keep Finding. Am J Respir Crit Care Med. 2017;195(6):714–5. Epub 2017/03/16. doi: 10.1164/rccm.201610-2019ED . 28294649 10.1164/rccm.201610-2019ED

[31] Dhooria S, Agarwal R, Gupta D. Is pirfenidone ready for use in non-idiopathic pulmonary fibrosis interstitial lung diseases? Lung India. 2015;32(1):4–5. Epub 2015/01/28. doi: 10.4103/0970-2113.148396 ; PubMed Central PMCID: PMCPmc4298916. 25624587 10.4103/0970-2113.148396 PMC4298916

[32] Dhooria S, Bal A, Sehgal IS, Aggarwal AN, Behera D, Agarwal R. Transbronchial lung biopsy with a flexible cryoprobe: First case report from India. Lung India. 2016;33(1):64–8. Epub 2016/03/05. doi: 10.4103/0970-2113.173066 ; PubMed Central PMCID: PMCPMC4748668. 26933310 10.4103/0970-2113.173066 PMC4748668

[33] Dhooria S, Sehgal IS, Bal A, Aggarwal AN, Behera D, Agarwal R. Transbronchial lung biopsy with a flexible cryoprobe during rigid bronchoscopy: Standardizing the procedure. Lung India. 2016;33(2):248–9. Epub 2016/04/07. doi: 10.4103/0970-2113.177463 ; PubMed Central PMCID: PMCPMC4797458. 27051127 10.4103/0970-2113.177463 PMC4797458

[34] Dhooria S, Mehta R, Srinivasan A, Madan K, Sehgal IS, Pattabhiraman V, et al The safety and efficacy of different methods for obtaining transbronchial lung cryobiopsy in diffuse lung diseases. Clin Respir J. 2017:In Press. 10.1111/crj.12734 29105361

[35] Dhooria S, Sehgal IS, Aggarwal AN, Behera D, Agarwal R. Diagnostic Yield and Safety of Cryoprobe Transbronchial Lung Biopsy in Diffuse Parenchymal Lung Diseases: Systematic Review and Meta-Analysis. Respir Care. 2016;61(5):700–12. Epub 2016/03/05. doi: 10.4187/respcare.04488 . 26932382 10.4187/respcare.04488

[36] Ryerson CJ, Corte TJ, Collard HR, Richeldi L. A global registry for idiopathic pulmonary fibrosis: the time is now. Eur Respir J. 2014;44(2):273–6. Epub 2014/08/02. doi: 10.1183/09031936.00051914 . 25082902 10.1183/09031936.00051914

[37] King TE Jr., Bradford WZ, Castro-Bernardini S, Fagan EA, Glaspole I, Glassberg MK, et al A phase 3 trial of pirfenidone in patients with idiopathic pulmonary fibrosis. N Engl J Med. 2014;370(22):2083–92. Epub 2014/05/20. doi: 10.1056/NEJMoa1402582 . 24836312 10.1056/NEJMoa1402582

[38] Richeldi L, du Bois RM, Raghu G, Azuma A, Brown KK, Costabel U, et al Efficacy and safety of nintedanib in idiopathic pulmonary fibrosis. N Engl J Med. 2014;370(22):2071–82. Epub 2014/05/20. doi: 10.1056/NEJMoa1402584 . 24836310 10.1056/NEJMoa1402584

[39] Dhooria S, Sehgal IS, Agrawal R, Aggarwal AN, Behera D. Knowledge, Attitudes, Beliefs and Practices of Physicians Regarding Idiopathic Pulmonary Fibrosis and the Impact of a Continuing Medical Education Program. J Assoc Physicians India. 2017;65(11):30–6. 29322707

